# The Multifaceted Landscape of Myopia: Exploring Animal Models, the Optical and Visual Diet, Genetic and Environmental Factors, Access to Care, and Treatment Approaches

**DOI:** 10.1167/iovs.66.7.1

**Published:** 2025-06-05

**Authors:** Tina M. Winters, Molly Checksfield Dorries

**Affiliations:** 1 National Academies of Sciences, Engineering, and Medicine, Washington, District of Columbia, United States E-mail: twinters@nas.edu

In response to the tremendous increase in myopia globally over the past five decades, including a rise in the prevalence of high myopia and associated serious eye conditions and morbidities such as retinal detachment, the Board on Behavioral, Cognitive and Sensory Sciences (BBCSS) of the U.S. National Academies of Sciences, Engineering, and Medicine^1^ (the National Academies) initiated a consensus study to assess the current mechanistic understanding of myopia pathogenesis and causes of its increased prevalence, to identify knowledge gaps and barriers to progress, and to develop a research agenda aimed at better understanding of the biological and environmental factors that could explain its increasing incidence. BBCSS member Wilson (Bill) Geisler (University of Texas at Austin), along with his longtime collaborators Martin Banks (University of California, Berkeley) and David Williams (University of Rochester) helped to shape a statement of task for the study that called for attention to, among other topics, developing experimental studies to increase understanding of the link between risk factors for myopia development and the molecular and cellular mechanisms that contribute to eye growth, and identifying strategies to address barriers to the diagnosis and treatment of refractive error.

The National Academies appointed a committee of 15 researchers to carry out the study:

K. Davina Frick (Co-Chair), Johns Hopkins Carey Business School
Terri L. Young (Co-Chair), University of Wisconsin–Madison
Afua O. Asare, University of Utah
David Berson, Brown University
Richard T. Born, Harvard School of Medicine
Jing Chen, Rice University
Jeremy A. Guggenheim, Cardiff University
Anthony N. Kuo, Duke University
Daphne Maurer, McMaster University
J. Anthony Movshon, New York University
Donald O. Mutti, The Ohio State University
Machelle T. Pardue, Emory University
Ramkumar Sabesan, University of Washington
Jody Ann Summers, University of Oklahoma
Katherine K. Weise, University of Alabama at Birmingham

Collectively, the committee included expertise across a wide range of relevant fields and disciplines: vision science, visual neuroscience, ophthalmology, optometry, physical and physiological optics, experimental methodology, human factors, genetics, computer sciences, psychology, population studies, and healthcare delivery, organization, and financing. Information about the committee is available on the study website (https://www.nationalacademies.org/our-work/focus-on-myopia-pathogenesis-and-rising-incidence). The committee was appointed in April 2023 and over the course of about a year they met in person and virtually several times and worked collaboratively to develop a report that addressed their statement of task. The report, *Myopia: Causes, Prevention, and Treatment of an Increasingly Common Disease* was released in September 2024 and is available online to read or download in full free of charge (https://nap.nationalacademies.org/catalog/27734).

The study was supported by the National Eye Institute, the American Academy of Optometry, the American Optometric Association, Health Care Alliance for Patient Safety, the Herbert Wertheim School of Optometry & Vision Science, University of California, Berkeley, Johnson & Johnson Vision, Reality Labs Research, Research to Prevent Blindness, and the Warby Parker Impact Foundation. In accordance with National Academies’ core values of independence, objectivity, rigor, integrity, inclusivity, and truth, the committee's work was conducted independently from sponsors, and the report underwent the organization's rigorous review process before its release.

To inform its work, the committee commissioned papers on five key topics: animal models of human myopia, genetic and environmental factors in myopia, access to myopia care, treatment of childhood myopia, and the optical and visual diet in myopia. These papers were of tremendous value to the committee, and members drew on the papers in developing report content. The papers do not appear in full in the published report, but the versions prepared for the study are available on the landing page for the report linked above. Because the papers were deemed so valuable for the field, study staff and committee members advocated for broader dissemination of them. Authors were given the opportunity to make revisions and expansions to their papers; these updated papers underwent the *IOVS* peer review process and are published here collectively.
